# Protein markers of cancer-associated fibroblasts and tumor-initiating cells reveal subpopulations in freshly isolated ovarian cancer ascites

**DOI:** 10.1186/1471-2407-12-359

**Published:** 2012-08-18

**Authors:** My Wintzell, Elisabet Hjerpe, Elisabeth Åvall Lundqvist, Maria Shoshan

**Affiliations:** 1 Department of Oncology-Pathology, Cancer Center Karolinska CCK R8:03 Karolinska Institutet, Stockholm, S-171 76, Sweden; 2 Department of Gynecologic Oncology, Karolinska University Hospital, Stockholm, S-171 76, Sweden

**Keywords:** Ovarian carcinoma, Dissemination ascites, Cancer-associated fibroblasts, Tumor-initiating cells, EMT

## Abstract

**Background:**

In ovarian cancer, massive intraperitoneal dissemination is due to exfoliated tumor cells in ascites. Tumor-initiating cells (TICs or cancer stem cells) and cells showing epithelial-mesenchymal-transition (EMT) are particularly implicated. Spontaneous spherical cell aggregates are sometimes observed, but although similar to those formed by TICs *in vitro*, their significance is unclear.

**Methods:**

Cells freshly isolated from malignant ascites were separated into sphere samples (S-type samples, n=9) and monolayer-forming single-cell suspensions (M-type, n=18). Using western blot, these were then compared for expression of protein markers of EMT, TIC, and of cancer-associated fibroblasts (CAFs).

**Results:**

S-type cells differed significantly from M-type by expressing high levels of E-cadherin and no or little vimentin, integrin-β3 or stem cell transcription factor Oct-4A. By contrast, M-type samples were enriched for CD44, Oct-4A and for CAF markers. Independently of M- and S-type, there was a strong correlation between TIC markers Nanog and EpCAM. The CAF marker α-SMA correlated with clinical stage IV. This is the first report on CAF markers in malignant ascites and on SUMOylation of Oct-4A in ovarian cancer.

**Conclusions:**

In addition to demonstrating potentially high levels of TICs in ascites, the results suggest that the S-type population is the less tumorigenic one. Nanog^high^/EpCAM^high^ samples represent a TIC subset which may be either M- or S-type, and which is separate from the CD44^high^/Oct-4A^high^ subset observed only in M-type samples. This demonstrates a heterogeneity in TIC populations *in vivo* which has practical implications for TIC isolation based on cell sorting. The biological heterogeneity will need to be addressed in future therapeutical strategies.

## Background

Most patients with epithelial ovarian carcinoma (EOC) are diagnosed with stage III or IV disease, i.e., when dissemination is already at hand. Along with rapid development of chemoresistant disease, this brings down the 5-year disease-specific survival in EOC to 50% [1]. Dissemination is mainly abdominal via tumor cells in ascites. These are likely enriched for aggressive cells that may have exfoliated not only from an excised primary tumor but also from established and often numerous metastases.

Ascitic ovarian cancer cells are proposed to undergo epithelial-to-mesenchymal-transition (EMT) to a motile phenotype with low levels of E-cadherin and higher invasivity than the primary tumor cells [2,3]. They are thus similar to blood- or lymph-borne circulating tumor cells from other types of carcinoma. They are similar also in that they represent a less studied but phenotypically and pathologically progressed stage compared to the primary tumors.

Of importance for the present study, ascitic tumor cells may occur as single-cell suspensions and sheet-like cell aggregates, but occasionally also as compact spheres in which individual cells cannot be discerned. Although spheres have been associated with invasivity, e.g., in *in vitro* models using artificially created spheroids from cell lines [4-6], these *in vivo* populations have not been extensively characterized. Interestingly,”collective migration” of multicellular clusters of cancer cells has been described as an enhanced type of metastatic behavior in breast and colon cancer [7].

Sphere formation *in vitro,* dissemination and chemoresistance are in turn features associated with cancer stem cells or tumor-initiating cells (TICs). TICs are now regarded as a major cause of relapse in chemoresistant cancer, and thus need to be identified both for prognostic and therapeutic purposes [8-10]. In the experimental *in vitro* setting, TIC identity is commonly based on expression of two or three of a set of markers, and is verified based on high tumorigenicity and/or serial transplantation in nude mice. Irrespective of whether TICs originate from stem cells or from a certain subpopulation of cancer cells, they are regulated not only by genetic but also by epigenetic and adaptational events [11]. As part of adaptation to hypoxia, both TICs and normal stem cells depend on glycolysis rather than mitochondrial respiration for ATP. Importantly, hypoxia has been shown to promote tumor”stemness” as well as EMT [12,13], suggesting that in ascitic EOC spheres, the hypoxic interior might enrich for TICs.

Cancer-associated fibroblasts (CAFs) represent yet another parameter contributing to invasivity, metastasis and chemoresistance [14]. Immunohistochemical analysis has shown absence of the CAF marker smooth muscle actin (α-SMA) in normal ovarian tissue, whereas the stroma of metastatic EOC tumors abounded in CAFs [15].

To explore the molecular pathology of EOC ascitic cell populations, we have here examined spheres and single-cell populations that were isolated directly from patients. The samples were compared in terms of expression of metabolic, EMT, TIC and CAF markers, and analysed by western blot in order to specifically assess the net outcome of genetic, epigenetic and post-translational effects. Importantly, freshly isolated human EOC ascitic samples were used rather than subcultured clones or cell lines, in which pheno- and genotypic drift is a wellknown problem. As metabolic marker we used mitochondrial β-F1-ATPsynthase, which reflects mitochondrial oxidative respiration and is downregulated at the mRNA level in cancer cells [16]. It has prognostic power on its own and in a ratio to Hsp60, i.e., as a bioenergetic cell (BEC) index shown to be indicative of low mitochondrial respiration and worse prognosis in, e.g., breast, lung and head-and-neck cancer [17,18]. We also examined the mitochondrial transcription factor A, TFAM, as it correlates with cellular mitochondrial content [19,20] and hence indirectly with oxidative metabolism. With regard to EMT and motility, we examined E-cadherin and vimentin as standard markers of epithelial and mesenchymal cells, respectively [7]. For CAF markers, we used α-SMA and the receptor for PDGFβ (PDGFβR) [14,15].

The significance and use of TIC markers requires some consideration. For cell sorting/isolation purposes, the surface proteins CD44, CD117 [10,21,22] and EpCAM [23,24] are convenient antibody targets. They also have intriguing dual functions as adhesion molecules as well as receptors [11]. In TICs isolated from primary breast cancer, shRNA-mediated knockdown of CD44 led to decreased expression of stemness-associated genes and to loss of tumorigenicity [25]. CD133 is another common TIC marker, including in EOC [26]. However, CD133 has been questioned as a marker of tumor-initiating capability in EOC [22,27], and there is uncertainty as to the roles of different splice and glycosylation forms and different antibody clones [28,29].

TICs are also characterized by transcription factors that are essential for stem cell self-renewal and pluripotency, and which include Nanog and Oct-4A [10,11,22]. Several studies have used combinations of surface and intracellular markers, e.g., CD44, CD117, Nanog and Oct-4 to characterize TICs derived from experimental EOC spheroids created *in vitro*[10]. The same report also used ABCG2, the membrane efflux pump which defines a side population enriched for TICs [30-32] and which contributes to chemotherapy resistance [33].

In summary, it is clear that the biology and molecular pathology of malignant ascites are of importance for the understanding and treatment of this deadly disease [6]. There is, however, still a dearth of information on ascitic cell subpopulations *in vivo*, i.e., isolated directly from patients. Based on such material and on protein marker profiles reflecting known phenotypic traits, our results indicate potentially very high levels of circulating TIC-type cells in malignant ascites and demonstrate that ascitic spheres and monolayer-forming cells are two distinct *in vivo* populations with different tumorigenic potential, based on protein markers. For the first time, the presence in malignant ascites of cancer-associated fibroblasts is also indicated.

## Methods

### Patients, ascites and cell culture

Malignant ascites were collected from 22 ovarian cancer patients (Table 1) at the Department of Gynecologic Oncology, Karolinska University Hospital. The study was approved by the Regional Ethics Committee of Stockholm (EPN 2009/1897-31/1) and samples collected with the consent of the patients.

**Table 1:** **Clinicopathologic characteristics of cancer patients (*n =*22)**

	**No (%)**
**Carcinoma diagnosis**	
Epithelial ovarian	13 (59)
Fallopian tube	2 (9)
Primary peritoneal	7 (32)
**FIGO stage**	
IIIC	16 (73)
IV	6 (27)
**Histologic subtype**	
Serous	19 (86)
Endometroid	1 (5)
Clear cell	1 (5)
Unclassified	1 (5)
**Grade of differentiation**	
2	2 (9)
3	15 (68)
Not stated	5 (23)
**Time of sampling**	
**During 1**^**st**^**line platinum-based therapy**	
Chemosensitive	2 (9)
Refractory	1 (5)
**Recurrence**	
**PFI < 6 months**	
Before start of 1^st^ course recurrence treatment	7 (32)
During chemotherapy period	12 (55)
**Type of cells**	
M-type only	13 (59)
S-type only	4 (18)
M and S	5 (23)
Median age at diagnosis 66.5 years	

PFI; platinum-free interval.

Cells in the ascitic fluid were pelleted, resuspended in PBS, and separated on a discontinuous gradient consisting of (bottom to top) Lymphoprep (Axis-Shield, Oslo, Norway), Lymphoprep/Krebs HEPES Ring solution 3:1 and Lymphoprep/Krebs HEPES Ring solution 1:2 (Krebs HEPES Ring solution: 137 mM NaCl, 5.4 mM KCl, 0.34 mM Na_2_HPO_4_, 0.35 mM KH_2_PO_4_, 8 mM MgSO_4_, 1 mM HEPES pH 7.4). Cells were then centrifuged at 1,500 × g for 20 min.

Tumor cells were collected at the interphase between the top and middle layer, washed and plated. When spheres were present, these were collected by aspiration the day after. We define spheres as compact spherical aggregates where individual cells cannot be discerned. Sheets/irregular aggregates of discernible and dispersible cells have not been included in this category. All cells, including the ovarian cancer cell line SKOV-3, which originates from malignant ascites, were kept at +37°C in 5% CO_2_ in RPMI1640 medium supplemented with 10% fetal calf serum, 1% penicillin-streptomycin, 2 mM L-glutamine (Nordic Biolabs AB, Täby, Sweden). Finally, for comparison with the physiological spheres from patients, artificial spheroids were made *in vitro* from freshly isolated tumor cells using previously described methodology [34].

### Western blot analysis and immunoprecipitation

Whole-cell lysates of monolayer-forming cell samples and spheres, respectively, were made either immediately or after a maximum of one passage. This rapid analysis is important for avoiding culture-induced phenotypic drift. Protease and phosphatase inhibitor cocktails (P2714 and P5726; Sigma-Aldrich Sweden AB, Stockholm, Sweden) were added freshly to RIPA lysis buffer (1% NP-40, 0.5% sodium deoxycholate, 0.1% sodium dodecyl sulfate, 0.004% sodium azide). Samples were loaded for gel electrophoresis at 30 μg/sample. Aliquots of the one and same SKOV-3 lysate were run on each gel as an internal control. After separation, proteins were transferred onto PVDF membranes, confirmed with Ponceau S staining. Membranes were blocked for 1 h in 5% milk/PBS-Tween or 4% BSA/TBS-Tween. All antibodies were diluted in blocking agent. Membranes were incubated overnight at +4°C with primary antibodies and then for 1 h at room temperature with the appropriate HRP-conjugated secondary antibody (see complete list of antibodies in Additional file 1: Table S1). Images were developed with Western Lightning Plus-ECL (PerkinElmer, Upplands Väsby, Sweden) and captured using Kodak M35 X-omat processor. Densitometry results are based on median signals from raw-data tif-format images, and normalized based on GAPDH levels and against the same SKOV-3 lysate present on each membrane.

For immunoprecipitation, whole-cell lysates were incubated with primary antibody to Oct-4A or SUMO-1 overnight at +4°C. Immunocomplexes were collected using Protein A/G PLUS-Agarose beads (Santa Cruz Biotechnology), and were then washed four times in lysis buffer and further analyzed by western blotting. See complete list of antibodies in Additional file 1: Table S1.

### Statistics

Densitometry data were analysed using the IBM SPSS statistics 20.0 software (IBM Corp. NY, USA). Shapiro-Wilk test was used to determine whether variables/groups were normally distributed. Between-group comparisons were performed using the Independent *t*-test or Mann–Whitney *U* test, when appropriate. Spearman’s Rank Order correlation was used to test association between variables. In all tests, a two-tailed *p*-value < 0.05 was considered significant, and *p*-values <0.05 or <0.01 are represented with one and two asterisks, respectively, in the figures. Relevant statistics are presented in Tables 2 and 3. Due to lack of material, two samples (one M- and one S-type) could not be analysed for expression of α-SMA and PDGFβ-R. This was taken into account in the statistical analyses.

**Table 2:** Statistical analysis of relative protein expression as based on densitometry

**A. Comparison of M- and S-type samples, using Mann–Whitney*U*test/Independent*t*-test**						
**Variable**	**Sample size (n) Sample type**		**Median (IQR)**^**1**^**Sample type**		**Mann– Whitney U**	**p-value Exact Sign. (2-tailed)**
	**M**	**S**	**M**	**S**		
β-F1-ATPsynth.	18	9	0.74 (0.76)	0.66 (0.86)	72.0	0.659
100 K	18	9	1.9 (9.6)	29 (33.15)	33.0	0.012 *
ABCG2	18	9	0.17 (0.63)	0.06 (0.26)	52.5	0.148
CD44	18	9	0.13 (0.20)	0.00 (0.00)	8.50	<0.001 **
E-cadherin	18	9	0.71 (5.2)	8.6 (7.2)	31.0	0.009 **
Nanog	18	9	0.52 (0.72)	0.92 (2.21)	70.5	0.605
Oct-4A	18	9	1.1 (3.0)	0.02 (0.48)	26.0	0.003 **
SUMO-Oct-4A	18	9	0.96 (1.0)	0.45 (0.80)	34.0	0.014 *
TFAM	18	9	0.77 (1.0)	0.51 (2.0)	79.0	0.929
Vimentin	18	9	0.69 (0.8)	0.05 (0.10)	11.0	<0.001 **
**Variable**	**Sample size (n) Sample type**		**Mean (± Std.deviation) Sample type**		**t-value**	**p-value Signif. (2-tailed)**
	**M**	**S**	**M**	**S**		
α-SMA	17	8	1.8(±1.1)	0.86(±0.30)	3.042	0.006^2^ **
EpCAM	18	9	0.90(±0.47)	0.87(±0.44)	0.130	0.897^3^
**Variable**	**Sample size (n) Clinical stage**		**Mean (± Std.deviation) Clinical stage**		**t-value**	**p-value Signif. (2-tailed)**
	**IIIC**	**IV**	**IIIC**	**IV**		
α-SMA	17	8	1.1(±0.67)	2.3(±0.46)	−2.401	0.006^2^ **

^1^ IQR: Inter-quartile range.

^2^ Equal variances not assumed.

^3^ Equal variances assumed.

**Table 3:** **All significant correlations**^**1**^**, using Spearman’s Rank Order correlation**

**Variables**		**Spearman’s correlation coefficient (*r***_***s***_**)**	**Significance (2-tailed)**
CD44	Vimentin	0.810	<0.001 **
E-cadherin	100 K	0.676	<0.001 **
E-cadherin	CD44	−0.528	0.005 **
E-cadherin	Vimentin	−0.692	<0.001 **
EpCAM	100 K	0.494	0.009 **
EpCAM	β-F1-ATPsynthase	0.700	<0.001 **
Nanog	EpCAM	0.694	<0.001 **
Nanog	β-F1-ATPsynthase	0.432	0.024 *
Nanog	100 K	0.425	0.027 *

^1^ All marker combinations were tested. Only those showing a significant correlation are shown.

## Results

### Some but not all ascitic cell samples contain spontaneously formed spheres

The clinicopathologic characteristics of the 22 patients are shown in Table 1. The majority was serous adenocarcinoma and all patients had advanced stage of disease (FIGO stages IIIC or IV). Three of the cases were newly diagnosed, of which one was platinum-refractory and two were chemosensitive, and 19 were in relapse.

**Figure 1:**
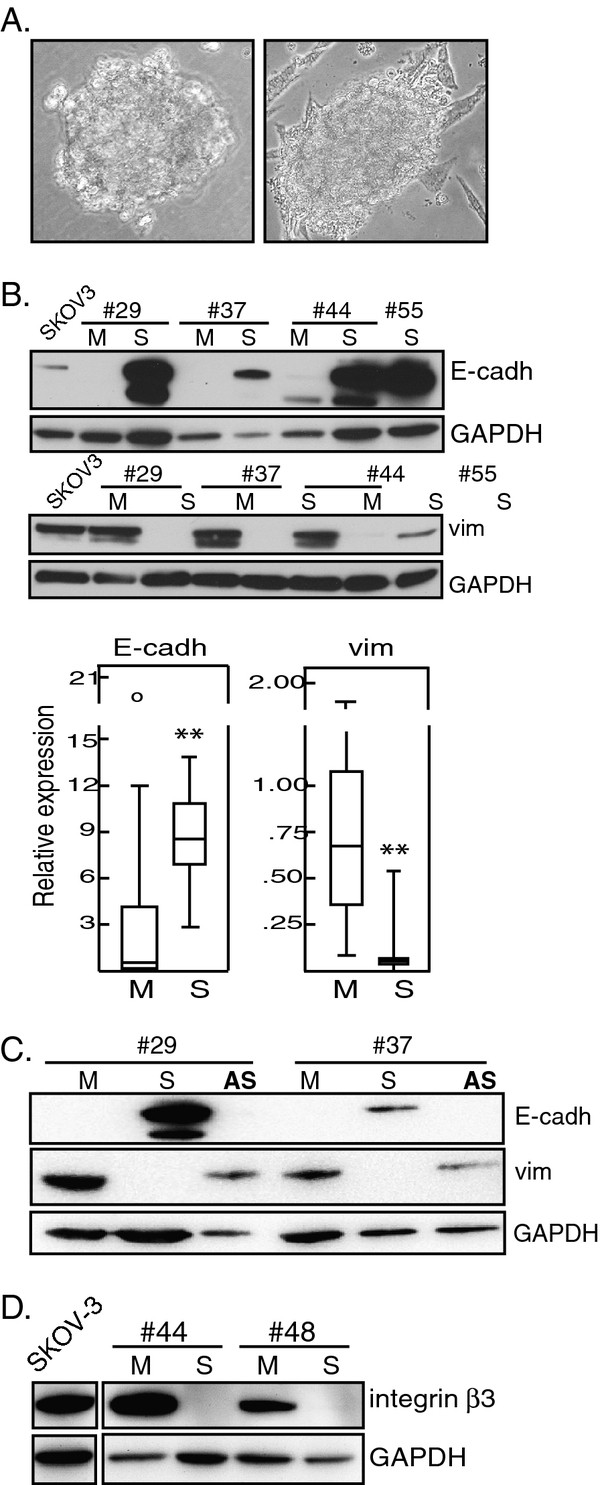
**Spheres constitute a separate population.** Ascitic cells were isolated from ovarian cancer patient ascites and separated into monolayer-forming single-cell suspensions (M-type samples; M) and spontaneous spheres (S-type samples; S), all as described in Materials and Methods. Lysates of each sample were analysed by western blot, with GAPDH as loading control. As interblot reference, a lysate (one and the same for all experiments) of the SKOV-3 cell line, derived from EOC malignant ascites, was used. Numbers refer to patients. **A.** Representative images of spheres, 40x magnification. *Left:* immediately after isolation. *Right:* Dispersed spheres do not form monolayers in culture, but continue to form spheres like this one and which then detach from the few adherent cells seen in the background. **B.***Top:* Representative example of E-cadherin and vimentin expression in paired M- and S-type samples, each pair from one and the same patient. *Below*: The data on E-cadherin and vimentin expression levels throughout the whole cohort are summarized in box plots comparing the distribution of relative expression of these proteins in all M (n = 18) and S (n = 9) samples, respectively. Asterisks denote statistically significant differences (Mann–Whitney *U* test). **C**. Representative examples of E-cadherin and vimentin expression in paired M- and S-samples from two patients, and artificial spheroids (**AS**) created *in vitro* using the M-sample cells. **D.** Expression of integrin β3 in two paired M- and S-type samples, each pair from one and the same patient.

The ascitic samples contained single cells, loose sheet-like aggregates and sometimes spheres (for definition, see Materials and Methods). Single-cell suspensions and easily dispersed loose aggregates readily formed monolayer cultures. Such samples are henceforth called M-type, and the isolated spheres are henceforth called S-type. In contrast to loose aggregates, the spheres could not be dispersed by pipetting, but only by passage through a fine-mesh filter. Moreover, the resulting cells would when plated sparsely never form monolayers but rapidly formed spheres again, often by a proliferative”budding” and detachment from a few adherent cells (Figure 1A). We conclude that the S-type cells are not senescent but proliferate as spheres rather than as monolayer cells.

Five patient samples contained both M- and S-type cells. These were prepared and analyzed as separate samples. Four patient samples contained only S-type. The study thus encompasses a total of 27 samples that are either M (*n* = 18) or S (*n* = 9).

### M- and S-type cells constitute separate populations

Western blots (exemplified in Figure 1B) and densitometry analysis of the whole material showed that E-cadherin expression was associated with S-type samples, but not with M-type (*p* = 0.009), and the mesenchymal marker vimentin with M- but not S-type (*p* = <0.001). These and all other statistics presented here are summarized in Tables 2 and 3.

To examine whether spherical growth per se was sufficient to induce E-cadherin, artificial spheroids were created from M-type samples, both from patient ascites with only M-type cells as well as from ascites containing both M- and S-type. The results showed that unlike the spontaneous S-type spheres, the artificial spheroids did not express E-cadherin (exemplified in Figure 1C).

Integrin β3 has been shown to be upregulated in EOC compared to normal ovarian surface epithelium [35] and has also been suggested as a prognostic marker [36]. Here, integrin β3 was assessed in two of the patient samples that contained both M- and S-type cells, and integrin β3 was expressed only in the M-type (Figure 1D). Altogether, these results show that S-type and M-type cells constitute two separate populations.

We initially hypothesized that due to internal hypoxia, spheres would show lower levels of the mitochondrial proteins β-F1-ATPsynthase and/or TFAM. This was refuted, however, as M- and S-type did not differ with respect to either protein (*p* = 0.659 and 0.929, respectively; Tables 2 and 3).

### Higher levels of CAF markers in M-type samples correlate with stage IV disease

**Figure 2:**
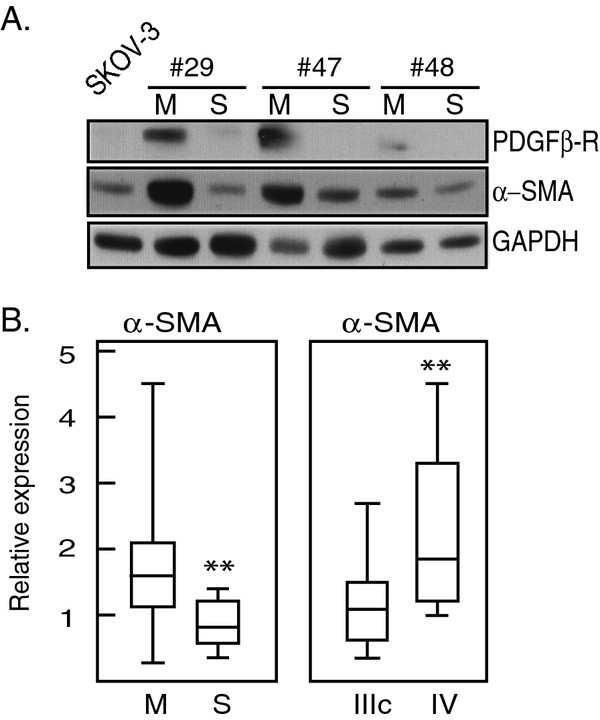
**Presence of cancer-associated fibroblasts in ascites**. A representative western blots showing co-expression of PDGFβR and α-SMA in paired M- and S-samples from three patients. In total 9/25 samples showed expression of PDGFβR, all of them M-type. Numbers refers to patients. **B**. Box plots comparing the distribution of relative expression of α-SMA in M- (n = 17) and S-type (n = 8) samples (*left*) and in samples representing clinical stage IIIC (n = 17) and IV (n = 8) at diagnosis (*right*). Asterisks denote statistically significant difference (Independent *t*-test).

Smooth muscle actin (α-SMA) and PDGFβ-R expression characterize activated cancer-associated fibroblasts [14]. Significantly higher levels of α-SMA were observed in M- compared to S-type samples (*p* = 0.006) (Figure 2A-B and Tables 2 and 3). Moreover, 9/25 samples analyzed showed PDGFβ-R expression, and all 9 were M-type (Figure 2A and not shown), and 8/9 samples expressing PDGFβ-R also expressed the ovarian cancer TIC marker CD44 and high levels of stem cell transcription factor Oct-4A (not shown and see below). 

Alpha-SMA was significantly higher in samples from patients diagnosed with stage IV compared to stage IIIC (p = 0.040). The clinical data (summarized in Table 1) were used for examining associations between markers and, e.g., time to progression, platinum-free interval, survival etc. We found that (*p* = 0.040) (Figure 2B; Tables 2 and 3). No other significant correlation was found (not shown).

### TIC surface markers: CD44 is expressed exclusively in M-type samples

**Figure 3:**
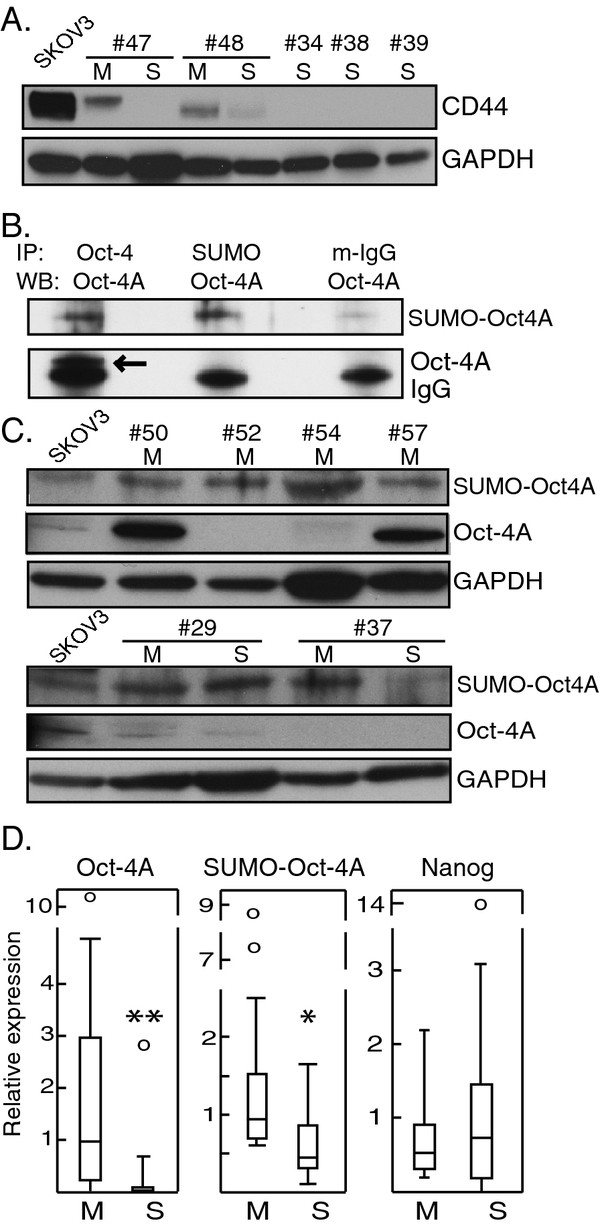
**TIC protein marker expression in M- and S-type samples. A**. Representative blot showing CD44 expression in M- and S-type samples. Based on all samples, CD44 was associated with M-type (*p*<0.001; Table 2). Numbers refer to patients. **B**. The SKOV-3 cell line was used for immunoprecipitation with antibodies to Oct-4 and to SUMO, respectively. Shown here is a representative western blot of three immunoprecipitates made with antibodies to Oct-4, SUMO and mouse IgG, respectively, and which were then probed using anti-Oct-4A. Mouse IgG served as negative control. SUMO-Oct-4A could be detected when probed with anti-SUMO (not shown). The results were confirmed using two other cell lines (not shown). Arrow: native ~40 kDa Oct-4A just above the 25 kDa IgG light chain. IP: immunoprecipitation; WB: western blot probe; m-IgG: mouse IgG.**C.** Oct-4A and SUMO-Oct-4A in M-type samples and paired samples of M- and S-type. **D.** Box plots comparing the distribution of relative expression of Oct-4A, SUMO-Oct-4A and Nanog, respectively, in all M (n=18) and S (n=9) samples. Asterisks denote statistically significant differences (Mann–Whitney *U* test).

The TIC marker CD44 [15,23,37,38] was found only in M-type samples (*p* < 0.001) (exemplified in Figure 3A; Tables 2 and 3). Accordingly, it correlated inversely with E-cadherin expression (*r*_*s*_ = −0.528, *p* = 0.005) and correlated strongly with vimentin expression (*r*_*s*_ = 0.810, *p* = <0.001) (Table 2). By contrast, M- and S-type did not differ with regard to TIC markers EpCAM [23,24] and ABCG2 [32] (*p* = 0.897 and 0.148, respectively; Table 2 and 3). CD117 was expressed in 4/27 samples and was not further analysed. 

### TIC transcription factors: Oct-4A and SUMO-Oct4A levels are higher in M-type samples

We examined expression of stem cell transcription factors Oct-4A and Nanog. Of the three isoforms of Oct-4, only Oct-4A is nuclear and crucial for stem cell renewal [39]. SUMOylation of Oct-4A is an important regulatory mechanism as it leads to increased stability and function of the protein [40]. SUMO-Oct4A has to our knowledge not been studied in ovarian cancer. We first identified the SUMO-Oct4A band in SKOV-3 lysates using immunoprecipitation of SUMO and Oct-4A, respectively, followed by western blotting for the same. A ~90 kDa band corresponded to SUMO-Oct4A (Figure 3B).

In the patient samples, SUMO-Oct4A was observed at varying levels in 24/27 samples while unmodified Oct-4A was seen in 17/27 samples (exemplified in Figure 3C). M-type samples showed higher expression than did S-type of Oct-4A and SUMO-Oct-4A (*p* = 0.003 and 0.014, respectively) (Figure 3D; Tables 2 and 3). By contrast, M- and S-type did not differ with respect to Nanog (*p* = 0.605) (Figure 3D; Tables 2 and 3).

There was a positive correlation between β-F1-ATPsynthase and Nanog (*r*_*s*_ = 0.432, *p* = 0.024; Tables 2 and 3), and between β-F1-ATPsynthase and EpCAM (*r*_*s*_ = 0.700, *p* = <0.001). Since Nanog and EpCAM also correlate (*r*_*s*_ = 0.694, *p* = <0.001), there is an association between these three markers, independently of sample type.

### A cellular 100 kDa fragment of E-cadherin in S-type samples

In addition to these transcriptional factors, we noted that in 13/27 samples E-cadherin was cleaved to a 100 kDa fragment which can be seen in Figure 1B and 1C, and which we here call 100K. As argued in the Discussion section, this is likely the calpain-cleaved form shown by others to have lost the β- and γ-catenin binding sites [41]. Such cleavage potentially leads to nuclear translocation of β-catenin and Wnt signaling involved in TIC maintenance [42]. The 100 K form was significantly higher in S-type samples than in M-type (*p* = 0.012) and correlated with expression of EpCAM (*r*_*s*_ = 0.494, *p* = 0.009), Nanog (*r*_*s*_ = 0.425, *p* = 0.027) and E-cadherin (*r*_*s*_ = 0.676, *p* = <0.001; Table 2 and 3).

## Discussion

Compared to experimental studies on tumor-initiating cells (TICs), the representation of TICs in the actual human *in vivo* situation is less well studied. This fact, and the nature of ovarian cancer ascitic cells as circulating and potentially metastatic cells, prompted us to examine freshly isolated ascitic samples for expression markers of EMT, TICs and cancer-associated fibroblasts (CAFs).

Previous studies have suggested that EOC ascitic cell spheres represent the invasive and/or metastasis-forming subpopulation leading to recurrent disease [4,6]. This notion has in part been supported by *in vitro* work on artificial spheroids, and is also based on the well-known fact that TICs can be isolated from *in vitro* spheroids cultured continuously in stem cell medium. Indeed, our own initial hypothesis was that the spontaneous EOC ascitic spheres are similar to TIC spheroids and that the hypoxic interiors of spheres would support or harbor TIC-like cells.

However, our results show that the S-type populations freshly isolated from patients were not only of an epithelial and thus less invasive phenotype, but they were also low in the EOC TIC marker CD44 and the stem cell transcription factor Oct-4A and did not express CAF markers. Compared to the M-type samples, the S-type thus present a less tumorigenic profile. It may be noted, however, that they likely represent a chemoresistant population since chemotherapeutic drugs do not penetrate such multicellular structures [34,43].

Among M-type samples, two subtypes could be discerned: one that was similar to S-type but obviously lacking some factor required for sphere formation in ascites, and a second subtype that was CD44^high^/Oct-4A^high^ and which in addition also expressed specific CAF markers. In addition to other reports on CD44 as an EOC TIC marker [10,21,22], the role of CD44 in regulating a TIC phenotype was recently shown when shRNA-mediated knockdown of CD44 caused breast cancer TICs to differentiate and abolished their tumorigenicity in mice [25]. Similarly, results of experimental knockdown of Oct-4 have indicated its role in regulation of a TIC phenotype [44].

We therefore propose that the second, CD44^high^/Oct-4A^high^ subtype of M is the more malignant one. This is based on the roles of CD44 and Oct-4A in defining TICs, on the motility/invasivity of vimentin-rich tumor cells, and on the expression of CAF markers α-SMA and PDGFβ-R.

In further support of this model, the inverse correlation which we observed between CD44 and E-cadherin may reflect the recent finding that in serial xenografts of EOC tumors, the tumorigenic CD44^high^ cells were low/intermediate in E-cadherin [45]. Moreover, CAFs may originate either as fibroblasts or from epithelial cells that have undergone EMT [14]. Here, the ratio of E-cadherin:vimentin in M-type samples and the adherence-independence of these cells *in vivo* are both in accordance with the latter scenario. Importantly, the observed co-expression of PDGFβ-R and α-SMA further strengthens the identity and presence of activated CAFs, whose role(s) as direct promoters of metastasis, angiogenesis and chemoresistance is increasingly clear [14,15,46].

In line with such roles, we found that samples with a diagnosis of clinical stage IV showed higher expression of α-SMA than did samples from stage IIIC patients (*p* = 0.040). This is in line with the recent finding that CAFs promote EOC growth and metastasis *in vivo*[15]. It is also in line with stage IV being defined by hematogenous metastasis to distant sites, e.g., to the liver. Altogether, this is to our knowledge the first report to indicate the presence of CAFs in EOC malignant ascites.

Transcription factors generally make up only a minute fraction of the entire cellular proteome, and TIC-associated transcription factors an even smaller fraction within a population. Yet we could easily detect Oct-4A and Nanog in the present material, indicating significant levels of TICs within the samples. It is likely of great importance for this result that the samples were not subcultured *in vitro*, where the differentiating conditions and the consequent asymmetric proliferation of TICs will soon dilute the levels of TIC markers.

We note that although Oct-4 was recently associated with advanced FIGO stage and higher histological grade in serous ovarian adenocarcinoma [47], the present report is the first to show high expression in EOC of Oct-4A, the isoform that is crucial for stem cell renewal [39]. We are hence also first to show high expression in EOC of the stabilized, and more active, SUMOylated form [48] of Oct-4A. Indeed, 24/27 (89%) of the samples expressed Oct-4A/SUMO-Oct4A. Moreover, and of importance for data interpretation in further studies, we found that in some samples virtually all Oct-4A was in the ~90 kDa SUMOylated form. This demonstrates that lack of ~40 kDa Oct-4A in a western blot does not necessarily correspond to lack of the protein. Interestingly, Ubc9 is the only SUMO-ligase required for SUMO-Oct-4A [48], and it is upregulated in ovarian cancer [49], although its prognostic potential has not been investigated.

The 100 K is likely the calpain-cleaved 100 kDa E-cadherin fragment that releases β-catenin allowing it to translocate to the nucleus and participate in Wnt-like signaling [41,50]. The antibody used here does not recognize any other known intracellularly cleaved fragment. Nor could the fragment be due to the well-studied extracellular cleavage of E-cadherin that generates the soluble extracellular form, sometimes known as gp80 or sE-cad, which would not be found in cell lysates nor be recognized by the antibody used. Moreover, the remaining cellular fragment from sE-cad is around 37 kDa, i.e., not the one we observed. It would be interesting to know whether 100 K is identical to the cytoplasmic E-cadherin observed at high levels in the multipotent, tumorigenic epithelial-mesenchymal hybrid subset of EOC tumors [45], and/or to internalized E-cadherin leading to increased β-catenin/Wnt signaling in EOC cells [51].

Like Oct-4A, Nanog is involved in stem cell renewal. In EOC, Nanog expression in tumors has been shown to correlate with clinical stage [52]. Here, Nanog correlated with TIC marker EpCAM (*r*_*s*_ = 0.694, *p* = <0.001) and with 100 K (*r*_*s*_ = 0.425, *p* = 0.027), but not with CD44 or Oct-4A. The results thus show that TIC surface and transcriptional markers do not necessarily coincide. This in turn suggests that while abundant reports show that cell sorting based on CD44 will enrich for TICs, there may *in vivo* exist several subsets of TICs. Applied to the present material, the model also suggests that the Nanog^high^/EpCAM^high^ samples represent a particular TIC subset which may be either M- or S-type, and which is separate from the CD44^high^/Oct-4A^high^ subset observed only in M-type samples.

## Conclusion

We have here demonstrated high expression of TIC markers in malignant ascitic cells, and thereby potentially high levels of TICs in ascites. M-type populations expressing CAF markers are likely more aggressive or tumorigenic than the S-type. Furthermore, Nanog^high^/EpCAM^high^ samples represent a particular TIC subset which may be either M- or S-type, and which is separate from the CD44^high^/Oct-4A^high^ subset observed only in M-type samples. This may have practical implications for TIC isolation based on cell sorting, since using a particular set of markers may result in a particular subset of TICs. It may moreover explain why the literature can present different sets of markers to identify TICs. The results also imply a biological heterogeneity that will need to be addressed in future therapeutical strategies.

## Competing interests

The authors declare that they have no competing interests.

## Authors' contributions

MW did all the lab work and statistics and contributed to writing and revision. EH and EÅL contributed all clinical material, clinical data and medical expertise. MS conceived of the study and drafted the manuscript. All authors read and approved the final manuscript.

## Authors' information

Supported by

Swedish Cancer Society, grant #10 0476

Swedish Science Council, grant #K2011-54X-21748-01-6

Olle Engkvist Foundation

Radiumhemmet Foundation, grant #101441

Golje Memorial Foundation, grant #LA2010-0041

## Pre-publication history

The pre-publication history for this paper can be accessed here:

http://www.biomedcentral.com/1471-2407/12/359/prepub

## Supplementary Material

Additional file 1 **Table S1.** Antibodies used. Click here for file
