# Supplementary material for: Protein markers of cancer-associated fibroblasts and tumor-initiating cells reveal subpopulations in freshly isolated ovarian cancer ascites
Source: BMC Cancer. 2012 Aug 18;12:359. doi: 10.1186/1471-2407-12-359 (PMC3517779; doi:10.1186/1471-2407-12-359)
Supplement: Additional file 1 — Table S1. Antibodies used. [file 1471-2407-12-359-S1.pdf]

Supplementary Table.

Antibodies used

**Antibody (clone)**	**Host**	**Company**	**Dilution**
Western Blot			
α-smoot muscle actin	rabbit	GeneTex, Labinova, Upplands-Väsby, Sweden	1:1000
ABCG2 (BXP-21)	mouse	Abcam, Cambridge, UK	1:1000
ATP5B	rabbit	Sigma-Aldrich, Stockholm, Sweden	1:1000
CD44	rabbit	Sigma-Aldrich	1:1000
c-kit (Y145)	rabbit	Abcam	1:1000
E-Cadherin (36)	mouse	BD Transduction Laboratories, Erembodegem, Belgium	1:5000
EpCAM (B302(323/A3)	mouse	Abcam	1:200
GAPDH	rabbit	Abcam	1:2000
Integrin β3 (PM6/13)	mouse	Abcam	1:1000
mtTFA	rabbit	Abcam	1:500
Nanog	rabbit	Abcam	1:300
Oct-3/4 (H-134)	rabbit	Santa Cruz Biotechnology	1:400
PDGF receptor β (28E1)	rabbit	Cell Signaling Technology, In Vitro Sweden, Stockholm, Sweden	1:1000
Vimentin (V9)	mouse	Sigma-Aldrich	1:10 000
Anti-mouse-HRP	sheep	GE Healthcare, Stockholm, Sweden	1:5000
Anti-rabbit-HRP	donkey	Abcam	1:2500
Immunoprecipitation			
Oct-4 (9B7)	mouse	Cell Signaling Technology	1:100
SUMO-1 (D-11)	mouse	Santa Cruz Biotechnology	1:125
